# Histological Architecture Underlying Brain–Immune Cell–Cell Interactions and the Cerebral Response to Systemic Inflammation

**DOI:** 10.3389/fimmu.2017.00017

**Published:** 2017-01-19

**Authors:** Atsuyoshi Shimada, Sanae Hasegawa-Ishii

**Affiliations:** ^1^Department of Pathology and Laboratory Medicine, Central Hospital, Aichi Human Service Center, Kasugai, Aichi, Japan; ^2^Department of Pharmacology, Pennsylvania State University College of Medicine, Hershey, PA, USA

**Keywords:** choroid plexus, leptomeninges, bone marrow transplantation, endotoxemia, cytokine, sepsis-associated encephalopathy, preterm brain injury

## Abstract

Although the brain is now known to actively interact with the immune system under non-inflammatory conditions, the site of cell–cell interactions between brain parenchymal cells and immune cells has been an open question until recently. Studies by our and other groups have indicated that brain structures such as the leptomeninges, choroid plexus stroma and epithelium, attachments of choroid plexus, vascular endothelial cells, cells of the perivascular space, circumventricular organs, and astrocytic endfeet construct the histological architecture that provides a location for intercellular interactions between bone marrow-derived myeloid lineage cells and brain parenchymal cells under non-inflammatory conditions. This architecture also functions as the interface between the brain and the immune system, through which systemic inflammation-induced molecular events can be relayed to the brain parenchyma at early stages of systemic inflammation during which the blood–brain barrier is relatively preserved. Although brain microglia are well known to be activated by systemic inflammation, the mechanism by which systemic inflammatory challenge and microglial activation are connected has not been well documented. Perturbed brain–immune interaction underlies a wide variety of neurological and psychiatric disorders including ischemic brain injury, status epilepticus, repeated social defeat, and neurodegenerative diseases such as Alzheimer’s disease and Parkinson’s disease. Proinflammatory status associated with cytokine imbalance is involved in autism spectrum disorders, schizophrenia, and depression. In this article, we propose a mechanism connecting systemic inflammation, brain–immune interface cells, and brain parenchymal cells and discuss the relevance of basic studies of the mechanism to neurological disorders with a special emphasis on sepsis-associated encephalopathy and preterm brain injury.

## Introduction

The central nervous system (CNS) used to be regarded as an immune-privileged organ (1). CNS immune privilege was experimentally defined by the fact that tissues that were rapidly rejected by the immune system when grafted in sites such as the skin, showed prolonged survival when grafted into the CNS (2). Isolation of the brain from the immune system by the blood–brain barrier (BBB) and the lack of draining lymphatics also contributed to the idea of brain immune privilege (3).

However, recent data have dramatically altered such a viewpoint by demonstrating that the CNS interacts actively with the peripheral immune system and is therefore immune competent. Interactions between the CNS and the peripheral immune system are achieved *via* humoral factors, nerve fibers, and cell–cell interactions under both non-inflammatory and inflammatory conditions. In this article, we will focus on the tissue architecture and cellular components that enable cell–cell interactions between the brain and the immune systems in organisms. We discuss how these interactions are relevant to neurological diseases in which systemic inflammation underlies the pathogenesis.

## New Aspects of the Brain–Immune System Interaction

The immune system plays an important role in maintaining the higher brain functions under non-inflammatory conditions. For example, severe combined immune deficiency mice and nude mice that are deficient in mature T cells exhibit cognitive deficits and behavioral abnormalities that are remedied by T cell restoration (4). Systemic depletion of CD4+ T cells leads to reduced hippocampal neurogenesis and impaired learning in the Morris water maze (5). Although there are no T cells present in the healthy brain parenchyma under non-inflammatory conditions, T cells accumulate in the meninges and express high levels of interleukin (IL)-4, which skews meningeal myeloid cells toward an anti-inflammatory phenotype during cognitive task performance (6, 7).

The so-called glymphatic system is a brain-wide perivascular pathway that consists of a paraarterial cerebrospinal fluid (CSF) influx route, a paravenous interstitial fluid clearance route, and an intracellular trans-astrocytic path that is mediated *via* aquaporin-4 (8, 9). The glymphatic system supports the exchange of CSF with interstitial fluid and plays a role in driving waste products by way of vectorial convective flow from the interstitium toward the paravenous space, where waste products may gain access to lymphatic vessels in the neck (8). Interestingly, natural sleep or anesthesia increases the volume of interstitial space by 60%, resulting in a remarkable increase in convective exchange of CSF with interstitial fluid, which propels the clearance of potentially neurotoxic waste products such as Aβ oligomers that accumulate during wakefulness (10).

More recently, functional lymphatic vessels that line the dural sinuses have been discovered by two groups independently (11, 12). These sinus-associated lymphatic vessels are able to carry both fluid and immune cells from the CSF (11). The dural lymphatic vessels absorb CSF from the adjacent subarachnoid space and brain interstitial fluid *via* the glymphatic system (12). Dural lymphatic vessels transport fluid into deep cervical lymph nodes *via* foramina at the base of the skull. Therefore, the dural lymphatic vessels and glymphatic system may act together to drive Aβ from the brain (13). In addition, the dural lymphatic system presumably provides architecture that supports immune cell dynamics during the performance of higher functions of the brain under non-inflammatory conditions. In this regard, impaired functions of the glymphatic system or dural lymphatic system may cause neurodegenerative dementia such as Alzheimer’s disease (AD).

## Sites of Intercellular Interaction Between Brain Cells and Immune Cells

### Bone Marrow Transplantation (BMT) to Search for the Brain–Immune Interface

Although the brain is now known to actively interact with the immune system under non-inflammatory conditions, the site of cell–cell interaction between brain parenchymal cells and immune cells remains an open question. It was reported that following BMT by intravenous injection of donor cells, donor-derived cells were distributed throughout the brain parenchyma, and differentiated into ramified microglia (14, 15). However, it is now recognized that postnatal hematopoietic progenitors do not significantly contribute to microglia renewal or homeostasis in the adult brain (16). Other investigators have claimed that the donors’ bone marrow-derived cells are found chiefly in association with blood vessels and are only rarely found in the brain parenchyma (17–19).

A novel BMT procedure has since been developed that is called “intra-bone marrow (IBM)-BMT” (20). In this procedure, BMCs are collected from the marrow of the donors’ long bones by perfusion, and the entire BMCs are injected directly into the bone marrow cavity of the recipients instead of being injected intravenously (20, 21). The advantage of this IBM procedure is that it facilitates the transplantation of mesenchymal stem cells (MSCs) as well as of hematopoietic stem cells (HSCs) from the donor into the recipient (22). Two to six days after BMT, higher numbers of both HSCs and MSCs were reported in the bone marrow cavity of chimeras prepared by using the IBM procedure than those resulting from a conventional intravenous procedure (23–28).

### Leptomeninges, Choroid Plexus Stroma, Perivascular Space, and Circumventricular Organs (CVOs) As Brain–Immune Interfaces

In our first set of experiments described in the present article that has been originally published in Ref. (29), we used bone marrow-chimeric mice prepared by the IBM procedure to investigate the distribution and time-dependent changes in the density of bone marrow-derived cells as well as in their differentiation in the brain under non-inflammatory conditions (29). Bone marrow-derived cells start to appear in the leptomeninges and choroid plexus stroma at 2 weeks after BMT, and to appear in the brain perivascular spaces at 4 weeks after BMT in the bone marrow chimera. In the leptomeninges, bone marrow-derived cells exhibit round or spindle-shaped morphology. Spindle-shaped cells are located along the pia and in close apposition to subpial astrocytic endfeet. Bone marrow-derived cells that have entered the perivascular space, which is a narrow space between the outer surface of capillary walls and the brain parenchymal surface, are situated in close apposition to perivascular astrocytic endfeet. In the choroid plexus stroma, bone marrow-derived cells with a round to ovoid morphology often appear in clusters. Isolated, scattered bone marrow-derived cells exhibit a spindle-shaped morphology without ramified processes. Importantly, leptomeninges, perivascular spaces, and choroid plexus stroma share tissue components that are contiguous due to the fact that these brain regions are derived from the same developmental origin. Therefore, these spaces are one of the major interfaces between the immune system and the brain.

At 1 month after BMT, bone marrow-derived cells enter structures that lack a BBB and that are collectively named CVOs, including structures such as the median eminence, posterior pituitary, subfornical organ, pineal gland, and area postrema. Most bone marrow-derived cells in the CVOs are closely associated with blood vessels and exhibit elongated cell bodies with rare ramified processes. Therefore, CVOs are also interfaces between the immune system and the brain.

### The Attachments of Choroid Plexus As a Novel Brain–Immune Interface

**Figure 1:**
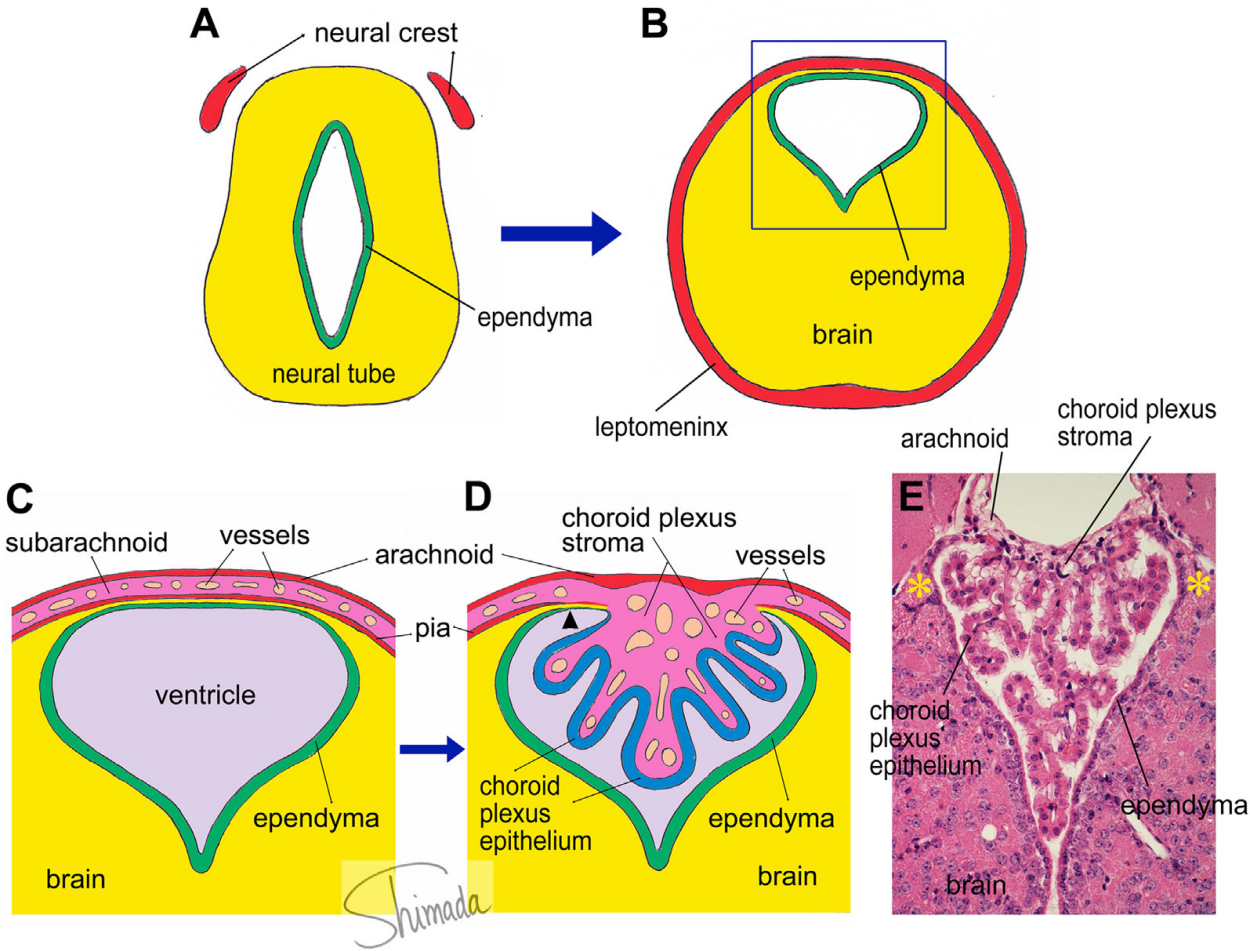
**Schematic illustration of choroid plexus morphogenesis**. **(A,B)** The neural tube contains the future cerebral ventricle and that the leptomenix is derived from the neural crest. In particular parts of the neural tube, the cerebral ventricle is shifted to the dorsal end of the brain parenchyma, from which the choroid plexus is formed. **(C)** An enlarged view of the square inset indicated in panel **(B)**. The choroid plexus is formed as a papillary protrusion of the subarachnoid stromal tissue **(D)**. Therefore, the choroid plexus stroma shares tissue components with the subarachnoid space and consists of loose connective tissue containing many capillary blood vessels. The choroid plexus epithelium is a continuation of the ependyma. At the edge of the choroid plexus, the brain parenchyma is extremely thin and located in a narrow channel between the ependyma and pia (arrow head). We call this particular structure the “attachments of choroid plexus.” The real histological architecture that is represented by the schema in panel **(D)** is indicated by a photoimage of a paraffin-embedded section of the mouse choroid plexus from the third ventricle stained with hematoxylin and eosin **(E)**. Note the presence of the “attachments of choroid plexus” in both sides (*).

A group of brain regions that has not been a focus of attention from scientists in the field is a group of particular brain parenchymal regions that is populated by bone marrow-derived cells (29). These regions are relatively small in size and discrete. Bone marrow-derived cells in these particular regions frequently exhibit multiple ramified processes (ramified marrow-derived cells). All ramified marrow-derived cells express Iba-1, a marker for myeloid cells (monocyte/macrophage lineage) (29). None of the ramified marrow-derived cells express markers for astrocytes, oligodendrocytes, or neurons. In chimeric mice in which the IBM procedure is used, most of these ramified marrow-derived cells start to appear at 4 months after BMT. The density of ramified marrow-derived cells increases thereafter, as a function of post-BMT time, for up to 8 months, which is the longest time point that we have examined so far (29). Ramified marrow-derived cells are distributed with the highest density (>16 cells/mm^2^) in the habenula and the brain stem cochlear nucleus, followed by the medial amygdala and the bed nucleus of stria terminalis (8–16 cells/mm^2^). They are distributed with moderate densities (4–8 cells/mm^2^) in the piriform cortex, thalamus, and cerebellar cortex. In the ventral hippocampus, superior colliculus, hypothalamus, and midbrain tegmentum, ramified marrow-derived cells are distributed at relatively low densities (2–4 cells/mm^2^). The regions populated by ramified marrow-derived cells contain junctions at which the choroid plexus is attached to the brain parenchyma (29). In the lateral ventricle, the choroid plexus is formed by papillary protrusion of the leptomeninges toward the inside of the ventricular cavity, where they bridge the stria terminalis and medial amygdala. The choroid plexus stroma shares tissue components with the leptomeninges and consists of loose connective tissue containing many capillaries. The choroid plexus epithelial cells are a continuation of the ependymal cells. At the edge of the stria terminalis or medial amygdala, the brain parenchyma is extremely thin and consists of loose wavy fibrous processes of astrocytes that are located in the narrow channel between the ependyma and the pia (Figure 1). We call this particular structure that consists of ependyma, loose glial tissue, and pia that connects the brain parenchyma and the choroid plexus the “attachments of choroid plexus.”

**Figure 2:**
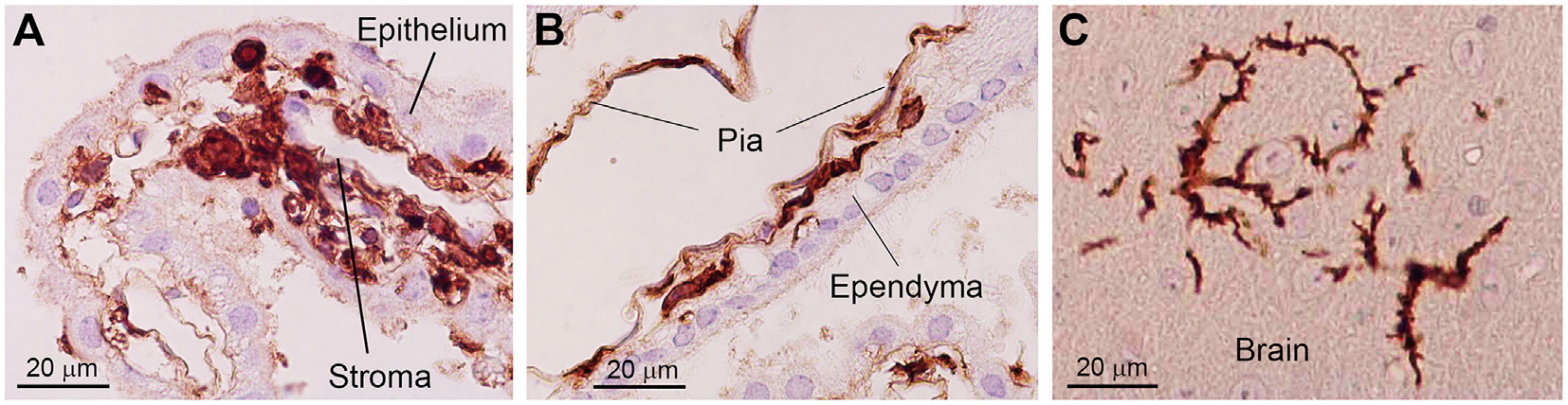
**Photoimages indicating changes in the morphology of bone marrow-derived cells in the brain**. In the choroid plexus stroma, bone marrow-derived cells exhibit a round to ovoid morphology **(A)**. When bone marrow-derived cells are located inside the attachments of choroid plexus, they are spindle-shaped and often exhibit a tortuous appearance with sharp bends **(B)**. Once these cells enter the brain parenchyma, they differentiate into a ramified morphology **(C)**. Immunohistochemical staining with an anti-GFP antibody. Nuclei are counterstained with hematoxylin. This figure is a modification of copyrighted material permitted by Academic Press (29), License Id: 3917441104164.

**Figure 3:**
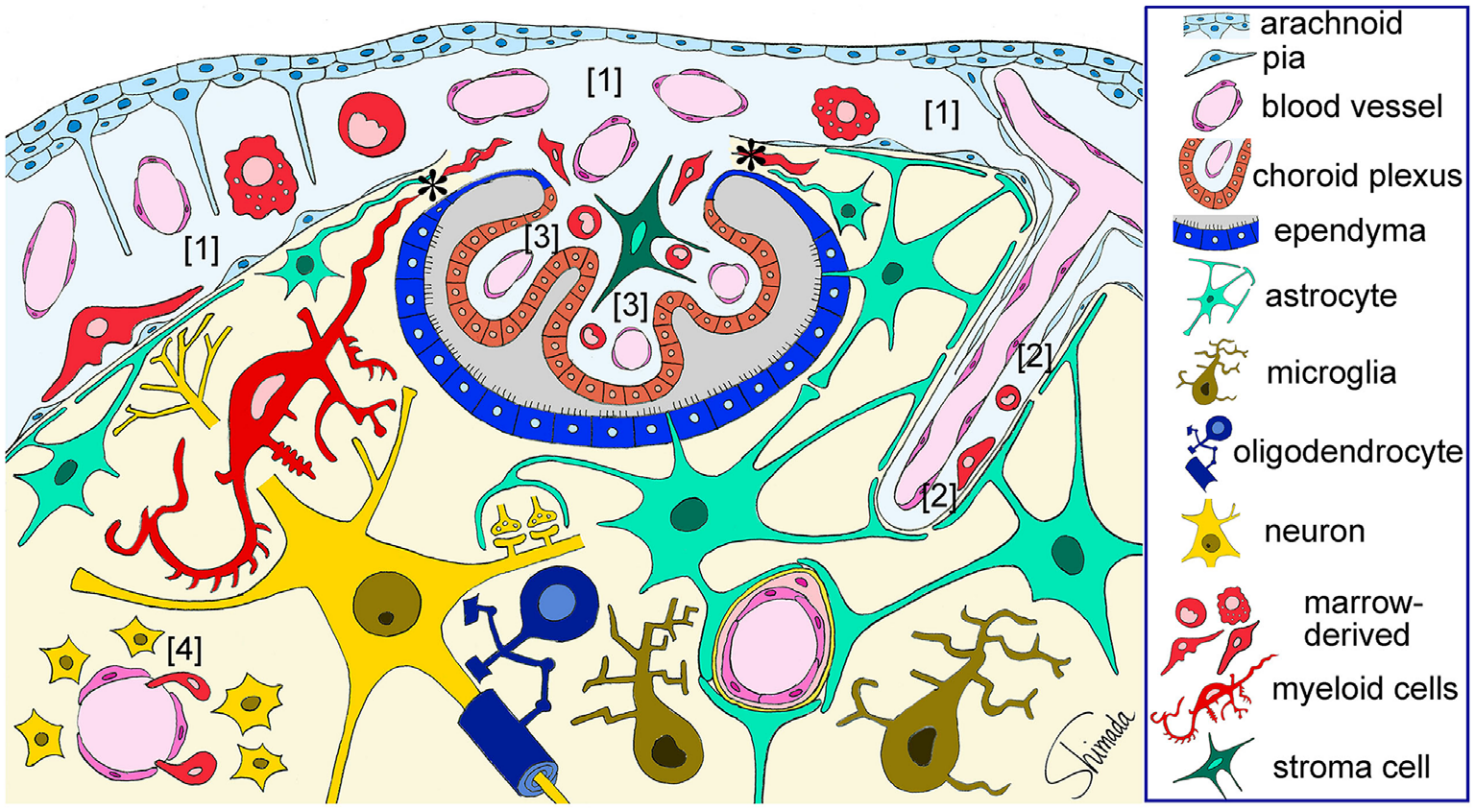
**Schematic illustration of the sites of brain–immune cell–cell interaction**. Red-colored cells represent bone marrow-derived myeloid cells that are located in the subarachnoid space [1], perivascular space [2], and choroid plexus stroma [3], all of which are contiguous. Bone marrow-derived cells enter the subarachnoid space through fenestrated blood vessels and can be localized in close apposition to the pia and subpial astrocytic endfeet that are a part of the brain parenchyma. Marrow-derived cells in the perivascular space can be localized in close apposition to perivascular astrocytic endfeet. Marrow-derived cells can cross the loose blood vessel walls of the circumventricular organs [4] and enter the parenchyma. Marrow-derived cells in the choroid plexus stroma can migrate into the brain parenchyma thorough the attachments of choroid plexus (*) without being blocked by the blood–brain barrier. These cells dramatically change their shape into a ramified morphology once they enter the parenchyma. Therefore, immune cells can interact with brain parenchymal cells *via* at least four types of histological architecture. This figure is a modification of copyrighted material permitted by Nature Publishing Group (31), License Id: 3917450680042.

Bone marrow-derived cells exhibit a round to ovoid morphology in the choroid plexus stroma, whereas bone marrow-derived cells are spindle-shaped and often exhibit a tortuous appearance when they are inside the attachments of choroid plexus. Once these cells enter the brain parenchyma, they cluster in a relatively small area and differentiated into ramified morphology (Figure 2) (30). These observations raise the possibility that bone marrow-derived cells enter the brain parenchyma through the attachments of choroid plexus. The sites of intercellular interaction between brain cells and immune cells that are based on the results of our IBM-BMT studies are summarized in a cartoon illustration in Figure 3.

There are presumably some molecular cues that enhance the migration of bone marrow-derived cells from the choroid plexus to the brain parenchyma. One such candidate cues is the CX3CL1–CX3CR1 signaling system. CX3CL1 (or fractalkine) is strongly expressed in the attachments of choroid plexus in both BM-transplanted and non-treated mice in a similar manner (30). CX3CL1 is constitutively expressed in a portion of the elongated cytoplasmic processes that emanate from astrocytes whose cell bodies are located in the adjacent brain parenchyma. Monocyte-derived cells in the choroid plexus and microglia in the brain parenchyma are known to express CX3CR1 (32), which is a highly specific receptor for CX3CL1 (33). CX3CL1 is a transmembrane molecule with a unique CX3C motif whose expression is induced by inflammatory cytokines (34). In addition to its functions as a cell adhesion molecule (33), membrane-anchored CX3CL1 can be released from the cell surface by cleavage and shedding mediated by A Disintegrin And Metalloproteinase (ADAM)10 and ADAM17 (35, 36). Once released, soluble CX3CL1 functions as a chemoattractant for cells bearing CX3CR1 (33, 34). The CX3CL1–CX3CR1 signaling system is also known to be required for physiological trafficking of circulating monocytes to peripheral organs such as lung (37) and small intestine (38). Therefore, bone marrow-derived cells that are in the process of lining up along the narrow channel of the attachments of choroid plexus may make cell–cell contact with astrocytic processes that express CX3CL1, resulting in their trafficking into brain parenchyma.

## Endotoxemia-Induced Cytokine-Mediated Responses of Hippocampal Astrocytes Transmitted by Cells of the Brain–Immune Interface

### Endotoxemia-Induced Impairments in Brain Higher Functions

The immune system modulates the functional and behavioral processes of the CNS under inflammatory conditions (39). Exposure to pathogens such as lipopolysaccharide (LPS) stimulates the peripheral immune system and induces inflammatory responses with the elevation of proinflammatory cytokines such as IL-1 (40, 41), IL-6, and tumor necrosis factor (TNF)-α (42). Increased levels of proinflammatory cytokines in peripheral tissues lead to the local synthesis of proinflammatory cytokines within the brain parenchyma (43–45), resulting in behavioral alterations such as sickness behavior (46–48), impaired learning (49), and depressive-like behavior in mice (50).

The hippocampus is one of the targets for behavioral changes induced by systemic inflammatory challenge. Types of memory tasks sensitive to peripheral LPS administration including a spatial water maze task (51), contextual fear conditioning (52), context discrimination memory (53), etc. are dependent on hippocampal function. However, LPS does not cross the BBB unless it is pathologically impaired (54). The amount of LPS entering brain parenchyma is only about 0.025% of an intravenously administered dose, which suggests that most effects induced by acute peripheral administration of LPS are not mediated through receptors expressed by brain parenchymal cells (55). Thus, how LPS outside the BBB mediates changes inside the brain is one of the most important remaining contemporary questions. Four possible routes by which the CNS and immune system might interact with each other have been proposed (56): a neural route *via* sympathetic or vagus nerves (57), CVOs, transport by cellular components that form the BBB (58), and secretion by vascular endothelial cells (58).

### Microglial Involvement in the Communication of the Immune System with the Brain

Microglia are known to be activated by systemic LPS administration (59). Intravenous injection of LPS (1–5 mg/kg) into rats induces a morphological transition of microglia to macrophage-like cells in the hypothalamus, thalamus, and brainstem 8–24 h after LPS injection (60). A systematic review of 51 studies that performed animal experiments has reported a relationship between systemic inflammation and microglial activation (61). Most studies have shown that microglia are activated time-dependently following single systemic challenge of mice and rats with LPS. What is required to induce CNS inflammatory responses during endotoxemia is the expression of toll-like receptor 4 (TLR4) on non-hematopoietic, intracranial resident cells located in the leptomeninges, choroid plexus and CVOs, cells along blood vessels, ependymal cells, and parenchymal microglia (62). Interestingly, non-parenchymal cells that are involved in sustained brain inflammation are located at the brain–immune interface as defined by our own studies using BMT (29, 30).

Depending on the experimental paradigm, activated microglia induced by systemic LPS administration can be neuroprotective. Daily intraperitoneal (i.p.) injections of LPS (1 mg/kg) for four consecutive days induce microglial activation that is detectable 24 h after the last injection, whereas microglial activation is not detectable at 24 h after a single i.p. injection of LPS (1 mg/kg) (63). Microglia thus activated by four daily i.p. injections of LPS are skewed toward an M2-like phenotype and participate in neuroprotection against experimental brain injury (63). It has been demonstrated that the activation of microglia induced by this LPS pretreatment paradigm is also independent of TLR4 on hematopoietic cells (63). Nevertheless, the mechanism connecting systemic inflammatory challenge and microglial activation has not been well documented (61).

### Importance of Astrocytic Endfeet in the Endotoxemia-Induced Hippocampal Responses

In our second set of experiments described in the present article that has been originally published in Ref. (31), we induced endotoxemia by a single i.p. injection of LPS at a dose of 3 mg/kg into mice, and by using Luminex multiplex assay technology, we identified cytokines that showed a change in concentration in the hippocampus and spleen. We then immunohistologically identified cells involved in the elevation of hippocampal cytokine levels (31). The concentration of at least 10 cytokines increases in the hippocampus in response to endotoxemia. Leptomeningeal stromal cells, choroid plexus stromal cells, and choroid plexus epithelial cells produce CC-motif ligand (CCL)2, CXC-motif ligand (CXCL)1, CXCL2, and IL-6 transiently at 4 h after LPS injection. Hippocampal vascular endothelial cells produce CXCL1 at 4 h after LPS injection. Importantly, these cells are located at the brain–immune interface but not in the brain parenchyma.

Cytokine whose concentrations are increased in the hippocampus at later than 4 h and at up to 24 h after LPS injection are produced chiefly by brain parenchymal astrocytes (31). Based on the fact that astrocytic endfeet express receptors for CCL2, CXCL1, CXCL2, and IL-6, it is considered that astrocytes are exposed to these cytokines *via* endfeet that are located in close apposition to cytokine-producing cells at the brain–immune interface. Stimulation of astrocytic endfeet with cytokines released by interface cells is very likely to result in inducing astrocytes to release another group of cytokines including CXCL10, CCL11, and granulocyte-colony stimulating factor (G-CSF) into the hippocampal interstitium (31). An increase in cytokines in the hippocampus occurs later than in the spleen, suggesting that the brain requires more steps than the spleen to produce cytokines in response to endotoxemia.

Astrocytes can produce CCL11 in response to endotoxemia. CCL11 in the brain is known to impair learning and memory (64), although this chemokine is known as a strong eosinophil chemoattractant that mediates allergic diseases. CCL11 also promotes the migration of microglia and induces microglia to produce reactive oxygen species, thereby enhancing excitotoxic neuronal death (65). Thus, astrocyte-derived CCL11 in our model may exert harmful effects on the hippocampal functions, leading to behavioral changes and memory impairment.

Astrocytes can also produce G-CSF in response to endotoxemia. Although G-CSF exerts neuroprotective actions on cerebral ischemic damage by promoting neuronal progenitor responses (66, 67), systemic injection of G-CSF into uninjured mice enhances the proliferation of microglia in the intact hippocampus (68). The latter observation suggests that G-CSF may act as a growth factor for microglia. Thus, astrocyte-derived G-CSF in our model may enhance the proliferation of microglia in the hippocampus, resulting in behavioral changes and memory impairment.

CXCL1 is produced by vascular endothelial cells. CXCR2, a receptor for CXCL1, is expressed on astrocytic perivascular endfeet. Given that cell–cell interactions with cytokines as mediators occur between cells located close to each other and that vascular endothelial cells are in close apposition to perivascular astrocytic endfeet that cover most of the perivascular surface of the cerebral microcirculation, a relationship between CXCL1 production by endothelial cells and CXCR2 expression by astrocytic endfeet appears reasonable. In the same manner, CCL2, CXCL1, CXCL2, and IL-6 are produced by leptomeningeal stromal cells, and the receptors for these cytokines are expressed on subpial astrocytic endfeet. A relationship between these cytokines and their receptors also appears to be reasonable given the close apposition between leptomeningeal cells and subpial astrocytic endfeet that line the entire brain surface.

In addition to these cell–cell interactions, choroid plexus epithelial cells may also release cytokines into the ventricular space and choroid plexus stromal cells may release cytokines into the subarachnoid space. A report in which the flow of subarachnoid CSF into and through the brain interstitium was investigated (8) has indicated that fluorescent tracers with low molecular weight (759 Da) infused in subarachnoid CSF move quickly throughout the brain interstitium. Tracers with intermediate molecular weight (3 kDa) infused in subarachnoid CSF concentrate in the paravascular space but also enter the brain interstitium to a limited extent from the paravascular space and from the pial surface. By contrast, large molecular weight tracers (2,000 kDa) infused in subarachnoid CSF are confined along paravascular space and do not enter surrounding interstitial space. Since the molecular weights of CCL2, CXCL1, CXCL2, and CXCL10 are approximately 7–8 kDa and that of IL-6 is approximately 18 kDa, expected movements of these cytokines in subarachnoid space are likely similar to those of tracers with intermediate molecular weight. Therefore, these cytokines may infiltrate the interstitium of paravascular and subpial regions.

Another possible pathway for cytokine flow from the choroid plexus into the hippocampus involves the attachments of choroid plexus (29, 30). Given that the hippocampus holds the attachments of choroid plexus at the fimbria, cytokines released by choroid plexus stromal cells may flow into the fimbria through the attachments of choroid plexus.

**Figure 4:**
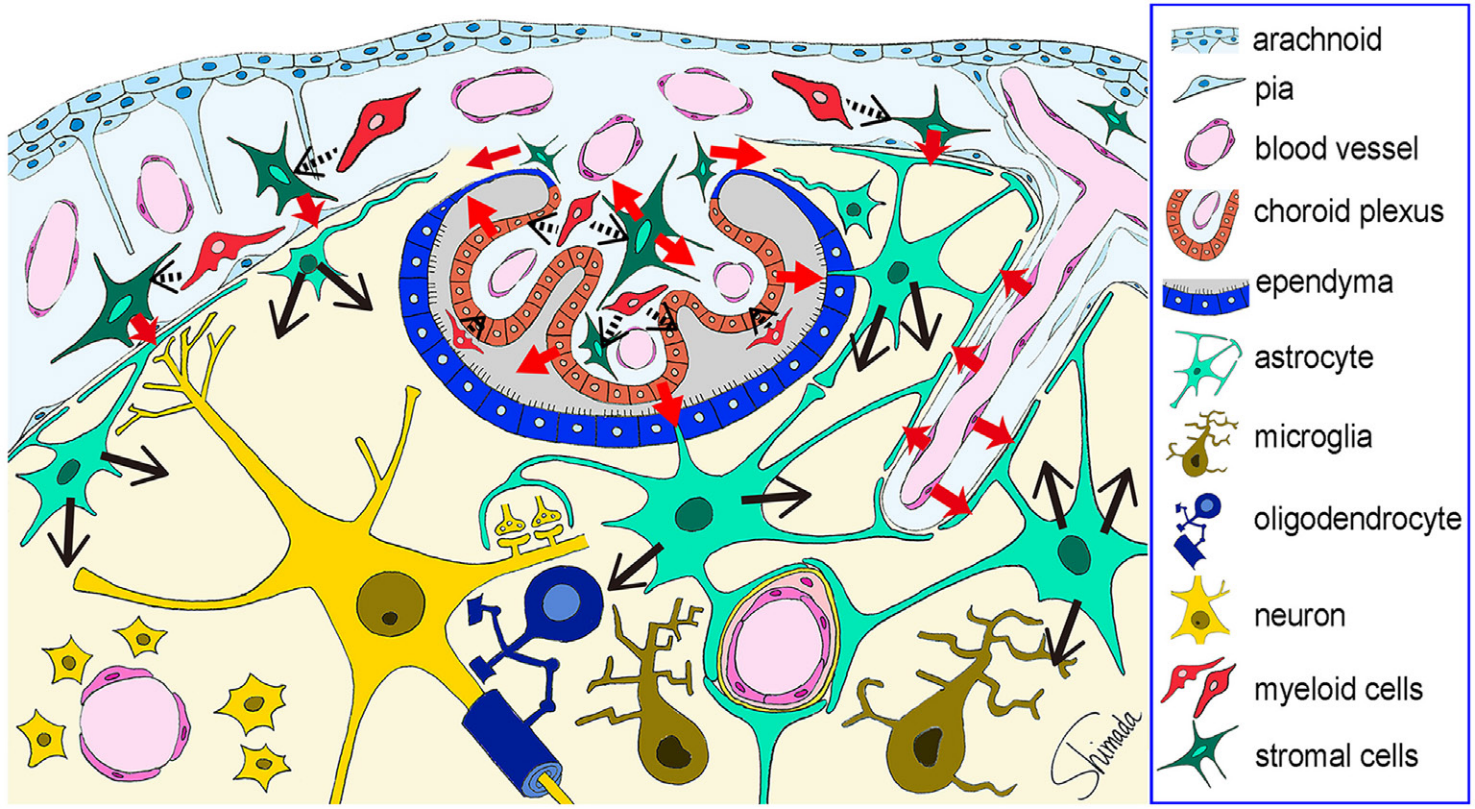
**Schematic illustration of the possible cytokine flow pathway by which systemic inflammation changes the brain cytokine microenvironment**. In response to systemic inflammation induced by intraperitoneal injection of lipopolysaccharide (LPS), hippocampal vascular endothelial cells, leptomeningeal stromal cells, choroid plexus stromal cells, and choroid plexus epithelial cells produce early cytokines (such as CCL2, CXCL1, CXCL2, and IL-6) at 4 h after LPS injection (*red arrows*). The receptors for these cytokines are expressed by astrocytic endfeet that are localized in close apposition to vascular endothelial cells and leptomeningeal cells. Thus, cytokine-mediated cell–cell interactions occur between the endothelial cells and the perivascular astrocytic endfeet, and between the leptomeningeal stromal cells and the subpial astrocytic endfeet. In addition, cytokines produced by choroid plexus stromal cells and epithelial cells may flow into the cerebrospinal fluid, and those produced by choroid plexus stromal cells may also flow into the brain parenchyma *via* the attachments of choroid plexus (*red arrows*). Thereafter, astrocytes produce late cytokines (such as CCL11, CXCL10, and granulocyte-colony stimulating factor) at 4–24 h after LPS injection (*black arrows*). An initial step in the activation of stromal cells in the leptomeninges and choroid plexus, and in the activation of choroid plexus epithelial cells, may be triggered by nearby myeloid cells, although this remains to be proven (*black arrows with dotted line*). This figure is a reproduction of copyrighted material permitted by Nature Publishing Group (31), License Id: 3917450680042.

Thus, cells of the brain–immune interface respond to endotoxemia with cytokine-mediated signals earlier than hippocampal parenchymal cells. In the parenchyma, astrocytes play a key role in responding to these signals by using endfeet with cytokine receptors that are located in close apposition to the interface cells. These results are summarized in a cartoon illustration in Figure 4. How the responses by astrocytes are relayed to microglia awaits further studies.

## Clinical Relevance of Basic Studies of the Structure and Function of the Brain–Immune Interface

### Perturbed Brain–Immune Interaction in a Wide Variety of Neurological and Psychiatric Diseases

Bone marrow-derived cells that enter the brain parenchyma increase in number in animal models of ischemic brain injury (69–71) and status epilepticus (72, 73), as well as in animal models of neurodegenerative diseases such as AD (74), Parkinson’s disease (PD) (75, 76), and amyotrophic lateral sclerosis (ALS) (77, 78). In experimental allergic encephalomyelitis, the choroid plexus is known as the CNS lymphocyte entry point (79). In a stroke model, bone marrow-derived perivascular, meningeal, and choroid plexus macrophages play an integral role in the inflammatory cascade (80). Repeated social defeat (81) and LPS-induced peripheral immune activation increases recruitment of bone marrow-derived monocytes into the brain (82). Recruitment of bone marrow-derived cells with the monocyte/macrophage lineage into the brain parenchyma is also enhanced in the senescence-accelerated mouse prone 10 (SAMP10), an animal model of human brain aging (83). The diencephalic structures located along the midline at the dorsal and ventral edges such as the choroid plexus epithelium, ependyma, astrocytic processes in the attachments of choroid plexus, periventricular astrocytes, tanycytes, and neurons of the median eminence and hypothalamus contribute to elevated tissue concentrations of CXCL1, CCL11, G-CSF, and CXCL10 in the brain. Changes in the cytokine profile accelerate the dynamics of immune cells migrating from the bone marrow to the diencephalon in SAMP10 mice.

Microglia are thought to be responsible for neuronal cell death in AD, PD, and ALS (84). Most published articles on microglia in these neurodegenerative diseases link activation of microglia with proinflammatory cytokine production (85). However, the presence of “dystrophic (senescent) microglia” in aged human brain has been proposed more recently (86). Given that microglia are neuroprotective, it is reasonable to consider that aging-related, progressive microglial degeneration, and loss of microglial neuroprotection rather than induction of microglial activation contributes to the onset of sporadic AD (86). This idea is also supported by our own findings on microglial aging in SAMP10 mice (87).

Proinflammatory status associated with cytokine imbalance is involved in a wide variety of psychiatric diseases such as autism spectrum disorders (ASDs) (88), schizophrenia (89), and depression (90). Autopsy findings of postmortem brains from individuals with ASD have indicated prominent microglia activation as well as increased production of inflammatory cytokines such as interferon (IFN)-γ, IL-1β, IL-6, TNF-α, and CCL2 in the brain tissue and CSF (88). Schizophrenia is associated with disruption of the cytokine milieu and the propensity for the production of proinflammatory cytokines (89). Acute psychotic episode is associated with increased serum concentrations of IL-6, TNF-α, IL-1β, and IFN-γ (89). Patients with major depression exhibit increased serum/plasma concentrations of IL-6 and C-reactive protein as well as elevated levels of IL-1β and TNF-α in peripheral blood and CSF (90).

Unlike multiple sclerosis, the archetypal inflammatory disease of the CNS in which an immune assault on the brain and spinal cord damages myelin sheaths and axons, most neurodegenerative and psychiatric diseases lack remarkable inflammatory cell infiltrates in the CNS (84, 91). Although monocytes and some other immune cells such as CD4+ and/or CD8+ T cells infiltrate the brain in neurodegenerative diseases, all of these immune cells appear in small numbers (84). Therefore, the involvement of immune abnormalities in aging and neurodegenerative diseases as well as psychiatric disorders is associated with imbalance in cytokine milieu rather than brain infiltration of inflammatory cells.

### Sepsis-Associated Encephalopathy (SAE)

Sepsis is the excessive systemic inflammatory reaction of an organism in response to an infection (92, 93). Septic encephalopathy or SAE is a multifactorial syndrome, which is characterized as diffuse brain dysfunction such as delirium, cognitive impairments, and loss of consciousness (94, 95). Importantly, patients develop SAE without clinical or laboratory evidence of direct brain infection or other types of encephalopathy (e.g., hepatic or renal encephalopathy) (94, 95). Instead, SAE is induced in the brain of an organism as a result of responses to systemic inflammatory changes. SAE is the most frequent type of encephalopathy encountered in the intensive care unit (92) and is present in about 20–30% of patients with sepsis. SAE often occurs before the failure of other organs (96) and, when present, it increases the mortality rate (97). In spite of the clinical importance of SAE, there presently exists no effective therapy that has been developed to specifically treat SAE in clinical settings (92), and only supportive therapy for the underlying disease is administered.

Although a case–control study in which the brain tissue of 13 patients with sepsis was compared with that of 17 controls reported a significant increase in the number of CD68-immunopositive activated microglia in the gray matter of septic patients (98), pathophysiological mechanisms of SAE have been proposed based chiefly on data obtained from experimental models. Disruption of the BBB due to loosened tight junctions of endothelial cells and detachment of the pericytes of hippocampal capillaries may play a role in SAE (99). Upregulation of intercellular adhesion molecule 1 during SAE (100) together with increased BBB permeability may induce circulating monocytes to adhere to the endothelium and pass into the surrounding tissues, where they proliferate and expand the population of perivascular macrophages (96).

In relatively early phase of sepsis, possible changes in the brain microcirculation may cause hypoxia–ischemia, which may lead to SAE through the upregulation of inflammatory gene transcripts including transcripts of TNF-α, IL-1β, and inducible nitric oxide synthase. TNF-α has been suggested to be a key inflammatory mediator. TNF-α can be produced intrinsically in the brain, it regulates aquaporin, and it alters the transport of water into the brain resulting in edema (101). Cytokines, together with reactive oxygen species and nitric oxide, can also mediate a decrease in mitochondrial ATP generation, resulting in an energy deficit in early sepsis (102–105). Another possibility is that there is an elevation in the levels of complement activation products during sepsis, which can cause brain tissue injury, and which, again, occurs *via* the release of cytokines (106).

Our experimental data indicating that cells of the brain–immune interface respond to endotoxemia with cytokine-mediated signals earlier than brain parenchymal cells and that parenchymal astrocytes play a key role in responding to these signals by using cytokine receptors on endfeet located in close apposition to the interface cells (31) would contribute to an understanding of the initial events leading to SAE.

### Preterm Brain Injury

Neonatal mortality and morbidity has been improved in the past half century. However, brain injury that occurs during the perinatal period is still a common cause of lifelong neurodevelopmental disabilities including cerebral palsy. The rates of cerebral palsy are not declining and may even be increasing in some western countries (107, 108). Therefore, the pathophysiology of perinatal brain injury remains a point of scientific debate. Although the etiology may be multifocal, hypoxia–ischemia, infection/inflammation, and excitotoxicity are considered important causes of perinatal brain injury (109). Experimental studies have indicated that antecedents such as infection/inflammation, intrauterine growth restriction, or preexposure to hypoxia modulate brain vulnerability (110, 111). Very recently, one of the mechanisms underlying the causal relationship between maternal immune activation and autistic behavioral abnormalities in the offspring has been revealed (112). Maternal immune activation increases IL-6 in the serum of pregnant mice, which leads to an increase in T_H_17 cells in maternal blood. These cells release IL-17, which crosses the placenta and increase the expression of IL-17 receptors in the offspring’s brain. This phenomenon in turn leads to autistic abnormalities in the offspring.

Brain injury evolves over time. Acute cerebral hypoxia/ischemia serves to cause depletion of tissue energy reserves, resulting in primary insult, which is often followed by transient restoration of energy metabolism upon reoxygenation. Thereafter, delayed or secondary cerebral energy failure parallels a decrease in tissue glucose metabolism and development of cell injury (113). Even after secondary cell death has subsided, effects on the brain persist including sensitization to inflammation or injury, increased seizure susceptibility, impaired oligodendrocyte maturation and myelination, and persistent inflammation and gliosis (114–116). Tertiary brain damage is defined in such situations as “injury caused by a long-persisting process following brain insult that worsens outcome, predisposes to further injury, or prevents repair/regeneration” (109). Tertiary brain injury underlies cerebral palsy (116), schizophrenia (117), and ASD (118).

An estimated 500,000 babies are born preterm (before 37 completed weeks of gestation) every year in the United States. Chorioamnionitis is a common cause of preterm birth. Clinical chorioamnionitis is characterized by maternal fever, leukocytosis, tachycardia, uterine tenderness, and prerupture of membranes (119), whereas subclinial chorioamnionitis is asymptomatic and is defined by inflammation of the chorion, amnion, and placenta (120). Subclinial chorioamnionitis is more common than clinical chorioamnionitis. Chorioamnionitis is often associated with a fetal inflammatory response syndrome that is defined by increased systemic inflammatory cytokine concentrations, funisitis, and fetal vasculitis (120). The fetal inflammatory response syndrome leads to a long-term adverse outcome such as impaired fetal cardiac function, chronic lung disease, retinopathy of prematurity, and lifelong neurological impairments including cerebral palsy, mental retardation, and learning deficits (120–123). Periventricular leukomalacia (PVL) is the predominant form of brain injury and the leading known cause of cerebral palsy and cognitive deficits in preterm infants (124).

Several animal models have been introduced for studies of preterm brain injury. The features, advantages, and disadvantages of each animal model of preterm birth are summarized elsewhere (125). Hypoxia–ischemia mouse, rat, and pig models and chronic hypoxia mouse and rat models have been utilized for the study of disorders in very low birth weight infants. The global mechanism by which perinatal hypoxia alters development is through a delay in the maturation of affected cell types, including astrocytes, oligodendrocytes, and neurons (125). Delays in oligodendrocyte maturation cause delayed myelination, which is typical of preterm white matter damage. By contrast, a proinflammatory status with cytokine imbalance has been reported in PVL (124, 126, 127). In this regard, LPS-induced inflammation mouse, rat, rabbit, and sheep models and an IL-1β administration mouse model are also useful for studies of preterm brain injury (125, 128).

An especially important characteristic of preterm newborns is that, when they develop systemic inflammation, they have the capacity to prolong the inflammation, resulting in intermittent or sustained systemic inflammation, and thereby increasing the risk of brain damage (129). Intermittent or sustained systemic inflammation contributes more to adverse neurodevelopmental outcomes in preterm infants than shorter duration inflammation (119). Therefore, future studies should address how prolonged systemic inflammation in preterm infants affects immature brains and causes long-lasting neurodevelopmental deficits. Since human babies at 25, 30, and 35 weeks of gestational age correspond to rodent pups at postnatal day 3 (P3), P7, and P10, respectively (125), newborn rodents treated with multiple systemic LPS injections can be a good model of intermittent or sustained systemic inflammation in preterm infants. The mechanism that connects systemic inflammation and microglial activation in preterm brains would be an especially promising avenue of research.

## Conclusion

The leptomeninges, choroid plexus stroma and epithelium, attachments of choroid plexus, perivascular space, CVOs, and astrocytic endfeet construct the histological architecture that provides a location for intercellular interaction between bone marrow-derived myeloid lineage cells and brain parenchymal cells under non-inflammatory conditions or at early stages of systemic inflammation during which the BBB is relatively preserved and the brain parenchyma exhibit no necrotic tissue damage. This histological architecture functions as the interface between the brain and the immune system to relay systemic inflammation-induced microenvironmental molecular events to the brain parenchyma. An understanding of how brain parenchymal cells, particularly microglia, respond to signals conveyed in this manner awaits further studies. It is essential that the mechanisms underlying the intercellular interactions among brain parenchyma cells as well as those between immune cells and brain cells be unraveled. Future investigations of these issues would enhance our understanding of the pathogenesis of SAE and of preterm brain injury.

## Ethics Statement

In our experiments described in the present article, all experiments were approved by the Animal Care and Use Committee of the Aichi Human Service Center. All mice were handled in accordance with the guidelines of Aichi Human Service Center and the Guide for the Care and Use of Laboratory Animals (National Academy Press, Washington, DC, USA).

## Author Contributions

AS designed the article. AS and SH-I contributed to the conception, writing, and editing of the manuscript. AS and SH-I carried out the experiments and analyzed the data in their studies described in the manuscript.

## Conflict of Interest Statement

The authors declare that the research was conducted in the absence of any commercial or financial relationships that could be construed as a potential conflict of interest.
